# A simple spreadsheet-based, MIAME-supportive format for microarray data: MAGE-TAB

**DOI:** 10.1186/1471-2105-7-489

**Published:** 2006-11-06

**Authors:** Tim F Rayner, Philippe Rocca-Serra, Paul T Spellman, Helen C Causton, Anna Farne, Ele Holloway, Rafael A Irizarry, Junmin Liu, Donald S Maier, Michael Miller, Kjell Petersen, John Quackenbush, Gavin Sherlock, Christian J Stoeckert, Joseph White, Patricia L Whetzel, Farrell Wymore, Helen Parkinson, Ugis Sarkans, Catherine A Ball, Alvis Brazma

**Affiliations:** 1 European Bioinformatics Institute, Wellcome Trust Genome Campus, Hinxton, Cambridge, UK; 2 Lawrence Berkeley National Laboratory, Berkeley, CA, USA; 3 MRC Clinical Sciences Centre, Faculty of Medicine, Imperial College Hammersmith Hospital Campus, London, UK; 4 Department of Biostatistics, John Hopkins School of Public Health, Baltimore, MD, USA; 5 Center for Bioinformatics and Department of Genetics, University of Pennsylvania School of Medicine, Philadelphia, PA, USA; 6 Department of Biochemistry, Stanford University School of Medicine, Stanford, CA, USA; 7 Rosetta Biosoftware, Rosetta Inpharmatics, LLC, Seattle, USA; 8 Bergen Center for Computational Science, Computational Biology Unit, University of Bergen, Norway; 9 Dana Farber Cancer Institute, Boston, MA, USA; 10 Department of Genetics, Stanford University School of Medicine, Stanford, CA, USA

## Abstract

**Background:**

Sharing of microarray data within the research community has been greatly facilitated by the development of the disclosure and communication standards MIAME and MAGE-ML by the MGED Society. However, the complexity of the MAGE-ML format has made its use impractical for laboratories lacking dedicated bioinformatics support.

**Results:**

We propose a simple tab-delimited, spreadsheet-based format, MAGE-TAB, which will become a part of the MAGE microarray data standard and can be used for annotating and communicating microarray data in a MIAME compliant fashion.

**Conclusion:**

MAGE-TAB will enable laboratories without bioinformatics experience or support to manage, exchange and submit well-annotated microarray data in a standard format using a spreadsheet. The MAGE-TAB format is self-contained, and does not require an understanding of MAGE-ML or XML.

## Background

### Introduction

Sharing of microarray data within the research community has been greatly facilitated by the development of disclosure and communication standards. The introduction of the Minimum Information About a Microarray Experiment (MIAME) standard [1] has been a great success, while the development of a common data representation format (MicroArray Gene Expression Mark-up Language (MAGE-ML), [2]) has enabled researchers to exchange data between laboratories and with public repository databases. However, the complexity of the MAGE-ML format has made its use impractical for laboratories lacking dedicated bioinformatics support. To address these needs, we propose a simple spreadsheet-based format for representing primary data and experimental details (metadata) from microarray investigations. We refer to this format as MAGE-TAB (MicroArray Gene Expression Tabular). Using MAGE-TAB, investigation design, array descriptions, and processed data are described by using tab-delimited files, or *spreadsheets *in the broad sense of the word. Additionally, the raw data, such as Affymetrix CEL or GenePix GPR files, can be provided in their native formats. Protocols are described using free text. Documents in this format can be created, viewed and edited in essentially any spreadsheet software (e.g. Microsoft Excel), which is typically familiar to biologists, who commonly use spreadsheets to maintain notes and track data. MAGE-TAB is designed for data collection and annotation, as well as for data communication between tools and databases, including submissions to public repositories.

**Figure 1:**
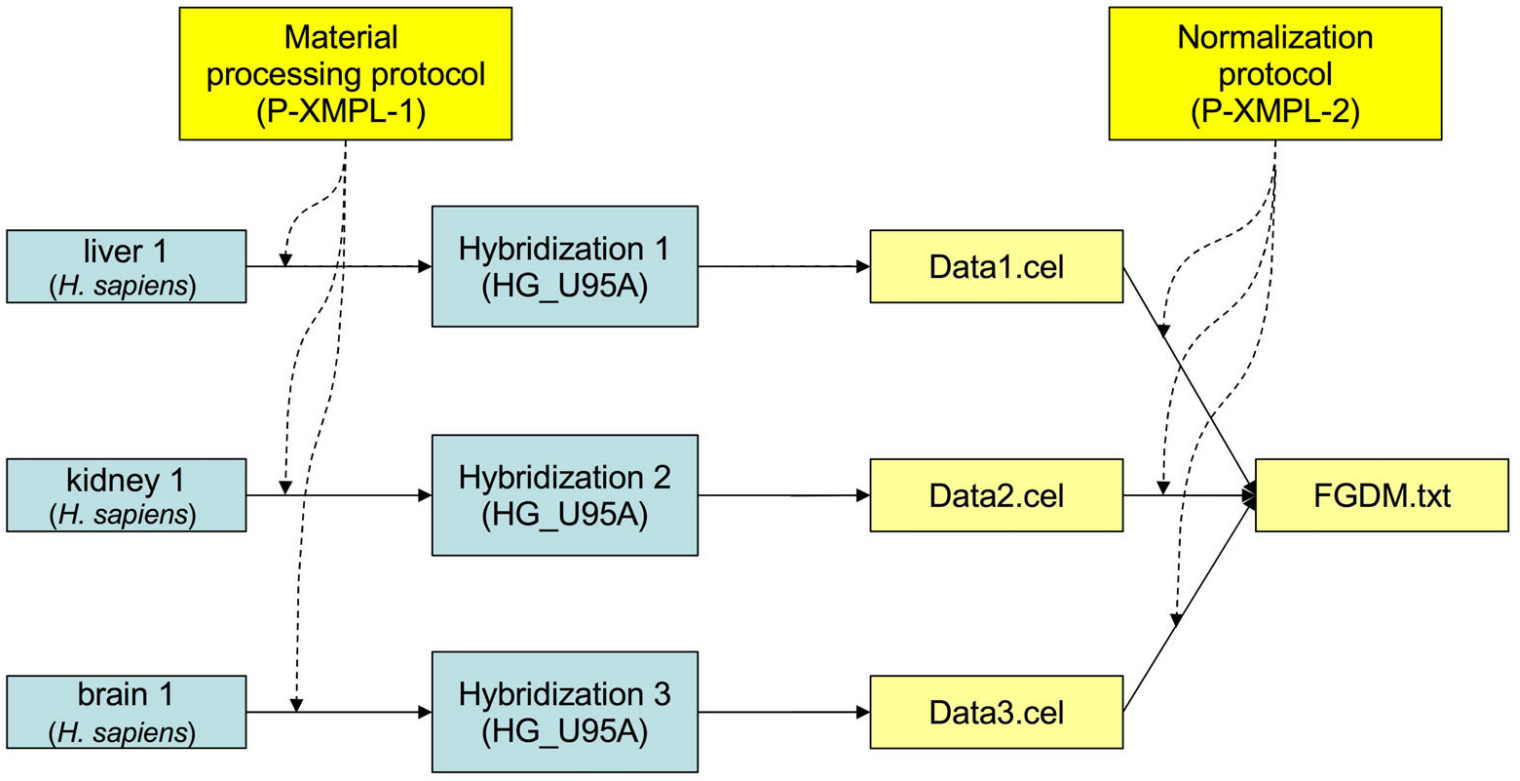
**An example of an investigation design graph for a simple one-channel array experiment**. Three samples are used: liver, kidney, and brain. Labeled RNA extracts from each sample are hybridized on an array (type HG_U95A). The RNA extraction and labeling are described in the Material processing protocol, P-XMPL-1. Raw data files (Data1.cel, Data2.cel and Data3.cel) are obtained, and then normalized and summarized as described in the Normalization protocol, P-XMPL-2, generating the file FGDM.txt.

One of the central concepts this paper will use to illustrate the proposed format is the *investigation design graph *(IDG). This IDG is a directed acyclic graph (DAG) representing relationships between samples (or more generally materials used in the investigation), arrays and data objects. An example of such a graph describing a simple one-channel microarray investigation is shown in Figure 1.

The information in this graph can be represented using a spreadsheet (or more generally a tab-delimited text file) in a simple and natural way, as shown in Table 1.

**Table 1:** A spreadsheet representation of the investigation design graph shown in Figure 1.

**Sample ID**	**Characteristics [Organism]**	**Characteristics [OrganismPart]**	**Protocol REF**	**Hybridization ID**	**ArrayDesign REF**	**ArrayData URI**	**Protocol REF**	**DerivedArrayData Matrix URI**
liver 1	Homo sapiens	liver	P-XMPL-1	hyb 1	HG_U95A	Data1.cel	P-XMPL-2	FGDM.txt
kidney 1	Homo sapiens	kidney	P-XMPL-1	hyb 2	HG_U95A	Data2.cel	P-XMPL-2	FGDM.txt
brain 1	Homo sapiens	brain	P-XMPL-1	hyb 3	HG_U95A	Data3.cel	P-XMPL-2	FGDM.txt

Each initial sample has a Sample ID (the first column in the spreadsheet) and Characteristics – Organism (genus and species) and OrganismPart (the second and third columns). The terms used to annotate the characteristics can be obtained from the MGED Ontology [26], another suitable source of controlled vocabulary terms, or provided as user defined terms. The fourth column gives a reference to a relevant protocol, while the fifth gives the IDs of the three hybridizations performed. The reference to the array design type (HG_U95A) is given as a hybridization property, which is followed by the data file names, a reference to the data normalization protocol and the normalized data file.

Although the example in Figure 1 and Table 1 relates to a one-channel experiment, two-channel experiments can be represented similarly, as demonstrated in the Implementation section below. The following principles guided the design of the MAGE-TAB format:

1. The format should be simple, but should also provide an explicit, structured representation of the details required by the MIAME standard.

2. The format should support concise description of the most frequently used experimental designs in a fashion familiar to biologists.

3. It should be possible to easily create, read, understand and edit documents in this format using only commonly available tools, and requiring no special training in bioinformatics or computer programming.

4. The format should have a formal definition, it should be machine-readable to the level of granularity defined by the MIAME structure, and it should be usable for communicating microarray data between different databases, data analysis tools and other software packages.

5. The formal definition should be based on the MAGE object model and for documents that can be expressed in MAGE-TAB there is a unique mapping to and from MAGE-ML. At the same time no general MAGE knowledge should be needed to use MAGE-TAB format.

The MAGE-TAB specification defines four different types of files to fully describe a microarray investigation:

1. Investigation Description Format (IDF) – a tab-delimited file providing general information about the investigation, including its name, a brief description, the investigator's contact details, bibliographic references, and free text descriptions of the protocols used in the investigation.

2. Array Design Format (ADF) – a tab-delimited file defining each array type used. An ADF file describes the design of an array, e.g., what sequence is located at each position on an array and what the annotation of this sequence is. If the investigation uses arrays for which a description has been previously provided, cross-references to entries in a public repository (e.g., an ArrayExpress accession number [3]) can be included instead of explicit array descriptions.

3. Sample and Data Relationship Format (SDRF) – a tab-delimited file (or files) describing the relationships between samples, arrays, data, and other objects used or produced in the investigation, and providing all MIAME information that is not provided elsewhere. This is often the least trivial part of the experiment description due to the complex relationships which are possible between samples and their respective hybridizations; however, for simple experimental designs, constructing the SDRF file is straightforward, and even complex loop designs can be expressed in this format.

4. Raw and processed data files – ASCII or binary files, typically in their native formats; alternatively, data may also be provided in a specially defined tab-delimited format termed a "data matrix", described below.

### Background and rationale

Microarray investigations can be interpreted only in the context of the experimental conditions under which the samples used in each hybridization were generated. Moreover, microarray data are highly dependent on the particular experimental and data processing protocols. This makes the use of microarray data considerably more complicated than, for instance, genome sequence data, and was the primary motivation for the development of the MIAME standard by the Microarray Gene Expression Data (MGED) Society [1]. As the title suggests, MIAME describes the data and metadata that authors must provide to support conclusions drawn from a microarray investigation, so that the data obtained in the investigation can be interpreted unambiguously and the investigation can be reproduced. The MIAME concept has been highly successful. It has not only guided the development of many software tools and databases, but has also been accepted by most of the major scientific journals as a means of making microarray data reported in publications available for scrutiny and for secondary analysis [4]. The fact that most journals now require MIAME-compliant data release as a condition of publication [5] has created a substantial data flow to public repositories. The two major databases, Gene Expression Omnibus (GEO) [6] and ArrayExpress [3], now house more than 150,000 hybridization experiments, representing over 5,000 investigations.

The main goal of MIAME is to make these data as useful as possible. MIAME provides the conceptual structure for the representation of microarray data including:

1. general information about the investigation and its design;

2. designs of the arrays used in the investigation;

3. characteristics of the samples used in the investigation;

4. experimental and data processing protocols;

5. raw and processed (normalized, filtered and/or selected) data.

An important concept in MIAME is that of the experiment or *investigation design *– the description of the relationships between different samples, arrays to which they have been hybridized, and the resulting data. Another important concept is that of *experimental factors*, such as time, dose, compound, or cell type. Experimental factors are typically the variables of interest in the investigation. Experimental factors enable the concise annotation of data – each column in the final data matrix can be annotated by the values of the most important factors characterizing the particular experimental conditions (e.g., the compound and the dose in a dose-response investigation). Logically, there may be one or more experimental factors per sample, where the samples for which all experimental factor values are identical are replicates. For more information about MIAME see [7].

Although MIAME identifies the details necessary to describe a microarray investigation, it does not provide a precisely defined format for data representation. Such a format is needed to cope with the growing flood of microarray data, and to facilitate automated communication between different microarray laboratory information management systems (LIMS), databases and data analysis tools. MAGE-ML was proposed by MGED in 2002 [2] and accepted as the Gene Expression specification standard by the Object Management Group [8]. It has been successful in that it has provided a platform for bioinformaticians and software engineers to understand and communicate information about high-throughput experiments in a precise language [9]. Moreover, many tools and databases have implemented MAGE-ML either fully or in part (e.g., [10-14]). Data generated by more than 20 different tools, representing more than 20,000 hybridizations, have been provided in the MAGE-ML format and deposited in ArrayExpress, and several fully automated data deposition pipelines have been established in both public and private organizations (e.g., [11])

MAGE-ML was the first nontrivial format developed for communicating high-throughput functional genomics experiment descriptions, and was developed at the same time as the community was first attempting the large-scale interchange of these functional genomic data. Nevertheless, MAGE-ML has not been accepted universally, for several reasons. One shortcoming is that MAGE-ML is ambiguous in that it permits encoding of the same semantic information in different ways (this was later addressed by developing the best practice 'How to encode MIAME in MAGE' document [15]). However, the main drawback has been the complexity of the MAGE-ML files, making it difficult to interpret or produce MAGE-ML files in the absence of a dedicated software development effort, which is seldom available for all but the largest laboratories.

In addition to MAGE-ML and MAGE-TAB, there are other specifications that support MIAME-compliant microarray data. From a repository perspective the most popular are the tab-delimited SOFT and the more recently introduced XML-based MINiML. These formats were introduced by GEO [6] and are designed to offer MIAME-capable data encoding with little object modeling expense. They are MIAME-supportive in the sense that they allow required information to be represented as free text descriptions. Free text is a powerful, highly flexible means for representing any information, but extracting the desired information – such as experimental factors and their values – may require some text mining. MINiML does not support structured, machine-parseable encoding of investigation design at the level of granularity required by MIAME; furthermore, the included free text descriptions are difficult to check for MIAME compliance, and not easily used in computation.

We believe that there is a need for a MIAME supportive format that is more structured than SOFT or MINiML, but offers a less complex implementation than MAGE-ML. With support from the National Human Genome Research Institute (NHGRI) and the National Institute of Biomedical Imaging and Bioengineering (NIBIB), the MGED members are developing the next generation microarray data standard, MAGE version 2 (MAGEv2 [16]). It has been proposed that MAGEv2 should consist of several inclusive layers of increasing complexity, where simple cases can be represented by simple constructs. The proposed MAGE-TAB format will be related to the simple layer of MAGEv2, and will be a sufficiently explicit, structured representation of information required by MIAME for almost all types of investigations. Although MAGE-TAB will be part of MAGEv2, it is important to note that no knowledge of object modeling is required to use MAGE-TAB and a substantially smaller set of documentation is necessary to learn MAGE-TAB.

## Implementation

### Investigation design graphs and their representations

**Figure 2:**
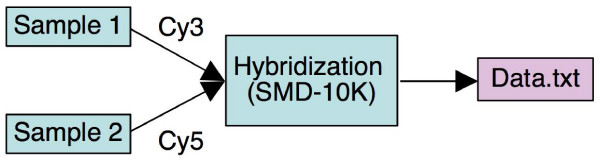
**An example investigation design graph**. This graph depicts two samples hybridized on an array (design name SMD-10K) labeled by Cy3 and Cy5, generating the data file Data.txt.

A key recommendation of the MIAME standard is the description of how biomaterials and data objects relate to each other within an experiment. Such relationships are most easily represented in graph form. A DAG in which nodes represent biomaterials (e.g., samples, RNA extracts, arrays) or data objects, and in which edges represent the relationships between these objects, can be represented as an IDG. For instance, an IDG can show which samples are hybridized on which array, producing which data files, as shown in Figure 2.

**Figure 3:**
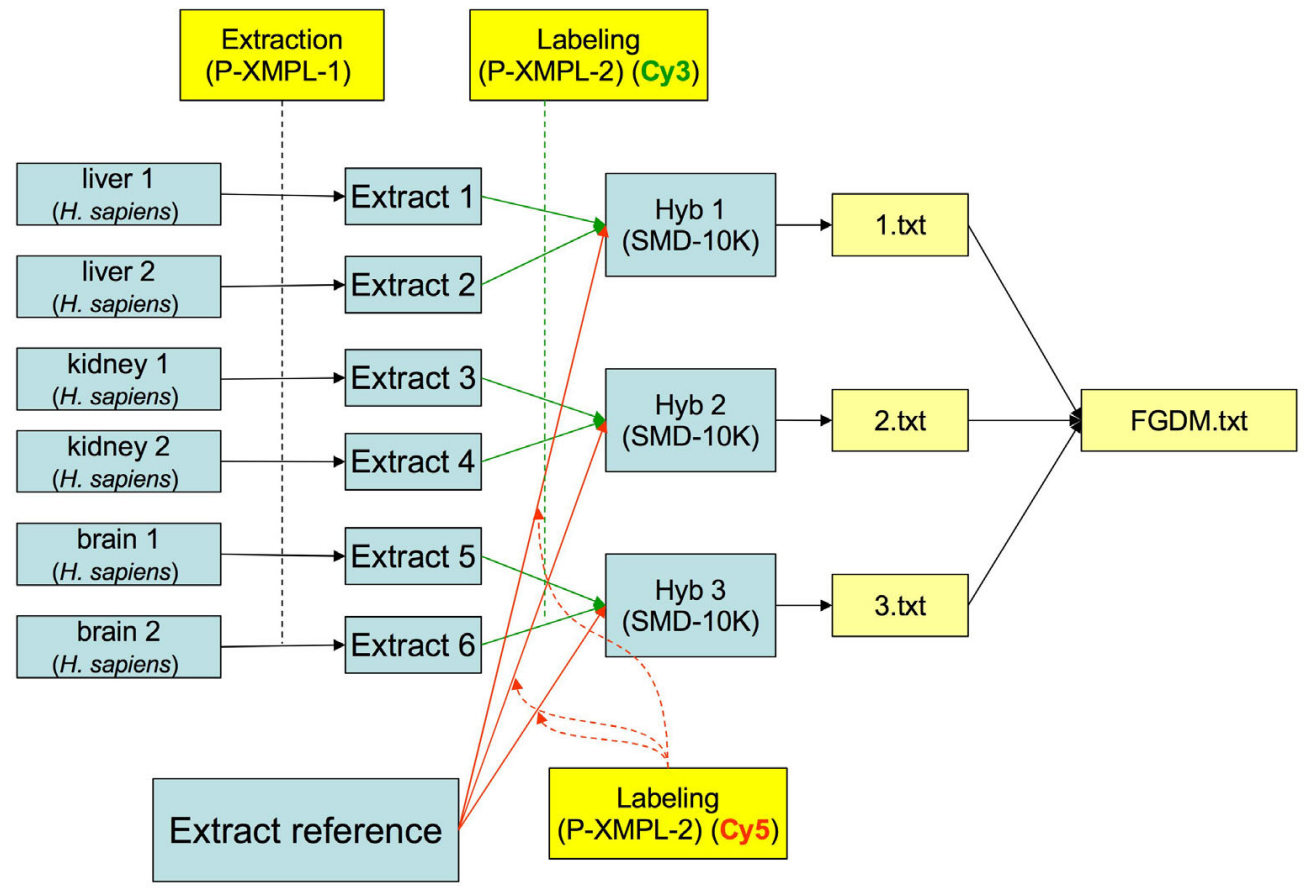
**An investigation design graph representing a two-channel experiment with extract pooling and reference RNA**. This investigation is similar to the example in the Introduction (Figure 1), except that it uses a two-channel array and an RNA reference. The extract pooling protocol has been omitted for clarity.

Nodes and edges in this graph can be annotated with information about the respective objects, such as sample characteristics. Edges (the relationships between nodes) can be annotated by pointers to the respective experimental or data processing protocols, or by protocol parameters (e.g., dyes Cy3 or Cy5 for labeling protocols). More complex investigation design graphs are shown in Figures 1 and 3. The IDG is a general concept applicable to any investigation description, and not restricted to microarray investigations. Effectively, the IDG represents the workflow of the investigation. The level of detail in this workflow description can vary; here we aim at the level of detail corresponding to the MIAME requirements. Two basic notions we use in defining the IDG are *biomaterial *and *data object*. The first intuitively represents a physical material such as a sample, RNA extract, array, or hybridized array. A protocol, when applied to a biomaterial, can generate a new biomaterial as its result. Biomaterials can also be split or pooled. For instance, one can take two samples, apply an RNA extraction/labeling protocol to each of them, labeling with Cy3 in the first case and with Cy5 in the second case, mix them and hybridize them on the array (as shown in Figure 2). Data objects can be created from biomaterials by applying a 'measurement' protocol, for example, by scanning a hybridized array to obtain feature intensities. Data objects can be transformed into new data objects by applying a data transformation protocol; for precise definitions of these objects MAGE-TAB will refer to the Functional Genomics Experiment (FuGE, [17,18]) object model that provides a higher-level class model for extension by technology-specific models such as MAGEv2 [16].

Each node in an IDG has an identifier and a list of labels. For instance, a node corresponding to a sample has the sample identifier and the sample properties, e.g., 'Organism' (genus and species) and 'OrganismPart' (organ). A label can be either a simple character string or a reference to an external object such as an ontology entry. For instance, 'Organism' will normally be described by an external ontology (e.g., NCBI taxonomy), 'OrganismPart' can be either a character string or an ontology entry obtained from an anatomy source of controlled terms. Edges in this graph can be labeled by protocols (or more usually by references to protocols) that have been used to derive one biomaterial from another. If protocols have parameters, these parameter values can be shown as labels on the respective edges (e.g., labeling protocols may have 'label' parameters, which can take values such as Cy3 or Cy5). Finally, each node in the graph has a *type*, e.g., 'sample', 'extract', 'hybridization', 'data'.

A question arises: How granular should the graph be? For instance, should one represent samples, extracts and labeled extracts within the same node, or using three different nodes? Note the differences between IDGs in Figures 1 and 3 – Figure 3 illustrates a two-channel experiment comparing a series of RNA extracts with a common reference extract, while Figure 1 represents a much simpler single-channel experiment. An additional layer of 'Extract' nodes have been used in Figure 3 to better indicate the point at which pooling occurred. In practice, the degree of granularity used in the IDG largely does not matter, unless one of the 'intermediate' objects is being split or pooled. Nodes in the graph that have only one incoming and one outgoing edge can be contracted into their predecessor nodes, by adding extra labels. Thus, unless extracts are pooled or split, it is sufficient to show which sample is hybridized to which array. Viewing a complex investigation design as a graph may be helpful, even if the graph is not drawn at the most granular scale possible. The graph representation makes the replicate structure in the investigation clear, and is even more valuable for developing software for data export/import from a database or tool. The possibility to represent an investigation design graph at different levels of granularity may seem to introduce ambiguity. However, the investigation design graph is an informal concept, and it is neither possible nor desirable to prescribe exactly how a particular investigation should be represented. For our purposes, the general guideline is that the graph should reflect the level of granularity defined by MIAME. We will show in the next section that this flexibility in the representation of an IDG does not substantially affect the investigation design representations in the resulting spreadsheets, as all these different graphs will result in essentially the same spreadsheet and encode the same semantic information. A labeled graph can be encoded in various ways; in MAGE-TAB, we use a tabular format for the following four reasons:

1. The observation that large investigation designs typically have a regular structure, i.e., the same sub-graph is repeated many times (possibly with well defined modifications); moreover, the replicated structure is simple. This observation was supported by analysis of the structure of over 1,000 different investigations in the ArrayExpress database.

2. The degree of nodes in these graphs (i.e., the number of incoming and outgoing edges for a node), is small (most often 1 to 3), except for a few specific nodes which are related 'reference' samples or extracts (e.g., 'Extract reference' in Figure 3).

3. The observation that DAGs which correspond to commonly used investigation designs have a property that their nodes can be grouped in consecutive layers, i.e., the source nodes (the nodes in the DAG which do not have entering edges) are in layer 1, the nodes that are connected to source nodes by an edge are in layer 2, etc. Furthermore, the grouping can be done so that each layer only contains objects of the same type, e.g., for the graph in Figure 3, we have sample layer 1, extract layer 2, hybridization layer 3, raw data file layer 4, and processed data layer 5.

4. Similar tabular formats have been used successfully in the biosciences and are familiar to many practitioners. For examples, see [19] for a spreadsheet approach to microarray data management, or [20], which describes the application of spreadsheets to the problem of data acquisition in the field of biochemical network modeling. In addition, the PRIDE database [21] is also developing a spreadsheet-based system for the submission of mass spectrometry data.

Once a DAG of a regular structure has been represented in such a layered fashion, it is natural to encode it as a tab-delimited file or 'spreadsheet'. Each node in a DAG is represented by entries in a contiguous set of columns within the spreadsheet. The first column within each set contains the ID of the node, with subsequent columns containing the labels attached to that node, followed by the labels of the edges leading from the node. Note that the labels in each list have a particular order. Objects of the same type (e.g., Sample, Hybridization, ArrayData) are all contained within the same column set, thereby capturing the layered DAG structure within the spreadsheet. Each row in the spreadsheet corresponds to a path in the graph from one of the source nodes to one of the 'sink' nodes. Thus if there are two or more edges leaving or entering a node, this node will appear in the spreadsheet once for each path passing through it. For instance, the DAG given in Figure 3 is represented as a spreadsheet in Table 2.

**Table 2:** SDRF representation of the investigation design graph in Figure 3.

**Sample ID**	**Characteristics [Organism]**	**Characteristics [OrganismPart]**	**Protocol REF**	**Extract ID**	**Protocol REF**	**Label**	**Hybridization ID**	**ArrayDesign REF**	**ArrayData URI**	**DerivedArrayData Matrix URI**
liver 1	Homo sapiens	liver	P-XMPL-1	Extract 1	P-XMPL-3	Cy3	Hyb 1	SMD-10K	1.txt	FGDM.txt
liver 2	Homo sapiens	liver	P-XMPL-1	Extract *2*	P-XMPL-3	Cy3	Hyb 1	SMD-10K	1.txt	FGDM.txt
kidney 1	Homo sapiens	kidney	P-XMPL-1	Extract 3	P-XMPL-3	Cy3	Hyb 2	SMD-10K	2.txt	FGDM.txt
kidney 2	Homo sapiens	kidney	P-XMPL-1	Extract 4	P-XMPL-3	Cy3	Hyb 2	SMD-10K	2.txt	FGDM.txt
brain 1	Homo sapiens	brain	P-XMPL-1	Extract 5	P-XMPL-3	Cy3	Hyb 3	SMD-10K	3.txt	FGDM.txt
brain 2	Homo sapiens	brain	P-XMPL-1	Extract 6	P-XMPL-3	Cy3	Hyb 3	SMD-10K	3.txt	FGDM.txt
				Extract reference	P-XMPL-3	Cy5	Hyb 1	SMD-10K	1.txt	FGDM.txt
				Extract reference	P-XMPL-3	Cy5	Hyb 2	SMD-10K	2.txt	FGDM.txt
				Extract reference	P-XMPL-3	Cy5	Hyb 3	SMD-10K	3.txt	FGDM.txt

Each 'layer' in the graph is represented by an ID column in the spreadsheet, followed by columns for each of the labels. Each path in the graph is represented by one row in the spreadsheet.

**Figure 5:**
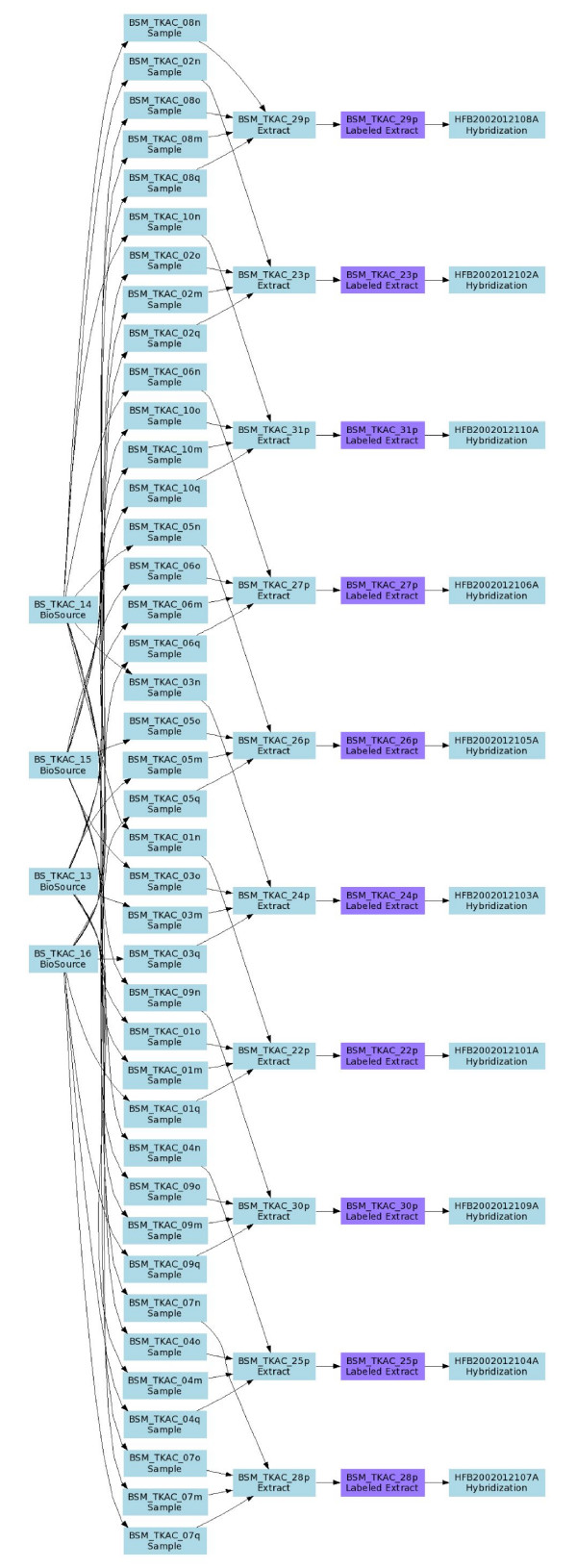
**An example of a more complex experimental design (data objects not shown)**. This is a real-world example, corresponding to the experiment with accession number E-MIMR-12 in Array Express.

Note that use of IDGs provides a powerful mechanism to describe the pooling or replicate structure of the investigation precisely and unambiguously. One can easily distinguish between biological replicates (different source nodes, but all having the same experimental factor values; see below for experimental factor definition) and technical replicates on various levels, such as several samples from the same source, or dye swaps (Figure 5).

### MAGE-TAB definition and examples

As described in the Introduction, a MAGE-TAB document includes four different types of files: (1) Investigation Description Format (IDF); (2) Array Design Format (ADF); (3) Sample and Data Relationship Format (SDRF); and (4) raw and processed data files. In this section we will describe each of these in more detail. Here we describe the main concepts and ideas upon which the format is based; the full MAGE-TAB specification is available online [22].

#### Investigation Design Format

An overall description of an investigation, including protocols and contact details, consists of a relatively small amount of information with few or no repetitious elements, and fits naturally into a single top-level document. Table 3 shows an example of an IDF document. Values for certain fields such as "Replicate Types" and "Protocol Type" may be drawn from the MGED Ontology [23] providing for a shared vocabulary of terms across files. Where fields may contain more than one term, these terms are separated using a semicolon delimiter.

**Table 3:** An example of an IDF.

**Investigation Title**	University of Heidelberg H sapiens TK6		
**Experimental Designs**	genetic_modification_design	time_series_design	
**Experimental Factors**	GeneticModification	Time	
			
**Person Last Name**	Maier	Fleckenstein	Li
**Person First Name**	Patrick	Katharina	Li
**Person Email**	patrick.maier@radonk.ma.uni-heidelberg.de		
**Person Phone**	+496213833773		
**Person Address**	Theodor-Kutzer-Ufer 1–3		
**Person Affiliation**	Department of Radiation Oncology, University of Heidelberg		
**Person Roles**	submitter; investigator	investigator	investigator
			
**Quality Control Types**	biological_replicate		
**Replicate Types**	biological_replicate		
**Date of Experiment**	2005-02-28		
**Public Release Date**	2006-01-03		
**PubMed ID**	12345678		
**Publication Author List**	Patrick Maier; Katharina Fleckenstein; Li Li; Stephanie Laufs; Jens Zeller; Stefan Fruehauf; Carsten Herskind; Frederik Wenz		
**Publication Status**	submitted		
**Experiment Description**	Gene expression of TK6 cells transduced with an oncoretrovirus expressing MDR1 (TK6MDR1) was compared to untransduced TK6 cells and to TK6 cell transduced with an oncoretrovirus expressing the Neomycin resistance gene (TK6neo). Two biological replicates of each were generated and the expression profiles were determined using Affymetrix Human Genome U133 Plus2.0 GeneChip microarrays. Comparisons between the sample groups allow the identification of genes with expression dependent on the MDR1 overexpression.		
			
**Protocol Name**	GROWTHPRTCL10653	EXTPRTCL10654	TRANPRTCL10656
**Protocol Type**	grow	nucleic_acid_extraction	bioassay_data_transformation
**Protocol Description**	TK6 cells were grown in suspension cultures in RPMI 1640 medium supplemented with 10% horse serum (Invitrogen, Karlsruhe, Germany). The cells were routinely maintained at 37 C and 5% CO2.	Approximately 10 cells were lysed in RLT buffer (Qiagen).Total RNA was extracted from the cell lysate using an RNeasy kit (Qiagen).	Mixed Model Normalization with SAS Micro Array Solutions (version 1.3).
**Protocol Parameters**	media	Extracted Product; Amplification	
			
**SDRF Files**	e-mexp-428_tab.txt		
			
**Database**	CTO	MO	nci_meta
**Database URI**			
**Database Version**		1.3.0.1	

The first column represents the qualifier name, while their values are given starting from column 2 (if the qualifier has two or more values, each is given in a separate column).

#### Array Design Format

The aim of the ADF component is to describe an array design in a spreadsheet or a set of spreadsheets. Conceptually, microarray designs are devised to measure presence and/or abundance of molecular (biosequence) entities in biological samples. Each sequence of interest is represented by one or more *reporter *sequences on the array, each of which in turn is present in one or more physical locations on the two-dimensional microarray surface. Thus three levels of hierarchy are required to describe the array design:

1. A *feature *on the array – a location (spot) on the array where nucleic acids are spotted or synthesized.

2. A *reporter *sequence – the sequence of the molecules present at a particular feature on the array. Note that the same reporter sequence can be present at different features, i.e., there is one-to-many relationship between reporter sequences and features.

3. A *composite element *– a set of reporter sequences designed to measure the same biological entity, such as a gene or an exon.

In the simplest case there may be a one-to-one relationship between reporter sequences and the biological objects they are measuring. However, in a more general case, there may be a set of reporters measuring the biological entity. For instance, on short oligonucleotide arrays (such as those produced by Affymetrix), many reporters are used to measure the expression of the same gene. In the most general case there may be a many-to-many relationship between the reporters and the biological entities they are measuring (for instance, the same short oligonucleotide may be present in several different splice variants of a gene). These concepts are derived from the MAGE object model. To describe a microarray layout fully, information about composite elements, reporter sequences, and features on the array, and the relationships (mappings) between them, must be provided. The ADF has been designed to provide the means to do this. An example of an ADF document is shown in Table 4.

**Table 4:** An example of an ADF document.

**Block Column**	**Block Row**	**Column**	**Row**	**Reporter ID**	**Reporter Sequence**	**Reporter Group**	**Control Type**	**CompositeElement ID**
1	1	1	1	R1	ATGGTTGGTTACGTGT	experimental		PTEN
1	1	1	2	R2	CCGCGTTGCCCCGCC	experimental		PAX2
1	1	1	3	R3	CGTAGCTGATCGATGA	experimental		WWOX
1	1	1	4	R4	GGTTGGCTGAGATCGT	experimental		MAPK8
1	1	2	1	R1	ATGGTTGGTTACGTGT	experimental		PTEN
1	1	2	*2*	R2	CCGCGTTGCCCCGCC	experimental		PAX2
1	1	2	3	R3	CGTAGCTGATCGATGA	experimental		WWOX
1	1	2	4	R4	GGTTGGCTGAGATCGT	experimental		MAPK8
...	...	...	...	...	...	...	...	...
4	6	20	20	462020	TCCCTTCCGTTGTCCT	control	control_spike_calibration	

Note how the information about Reporter and CompositeElement is duplicated to indicate the fact that every synthetic sequence is spotted more than once on the array.

#### Sample and Data Relationship Format

**Figure 4:**
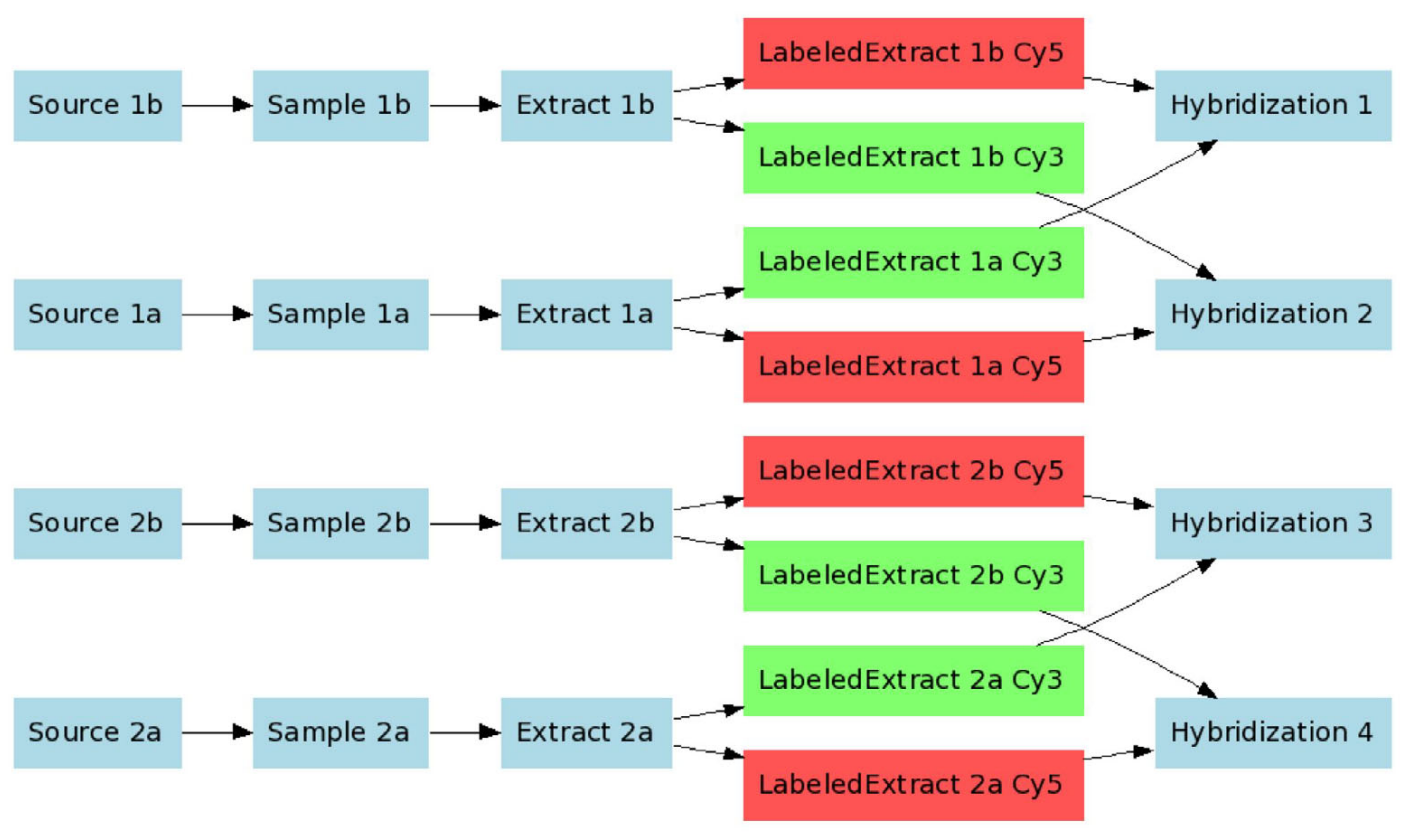
**Replicated design, dual channel with dye swap**. Data objects are not shown as there is a simple one-to-one mapping between hybridizations and raw data files.

The least trivial part of an investigation description is in the relationship between sample and data objects, as represented in the SDRF file. As already mentioned, an investigation design can be described as a DAG, and the SDRF is a spreadsheet-based representation of such graphs. Tables 1 and 2 show SDRF examples representing the investigation design graphs shown in Figures 1 and 3, respectively. Similarly, Figure 4 shows a simplified experimental design graph of replicated design, dual channel with dye swap (the protocols and data files are omitted for simplicity), and its spreadsheet representation is shown in Table 5. In the next example (Figure 5), Sources are split into Samples, which are then pooled into Extracts as shown. The IDG in Figure 5 can be represented by the SDRF in Table 6.

**Table 5:** Replicated design, dual channel with dye swap. Data objects have been omitted for brevity.

**Source ID**	**Sample ID**	**Extract ID**	**LabeledExtract ID**	**Label**	**Hybridization ID**
Source 1a	Sample 1a	Extract 1a	LabeledExtract 1a Cy3	Cy3	Hybridization 1
Source 1b	Sample 1b	Extract 1b	LabeledExtract 1b Cy5	Cy5	Hybridization 1
Source 1a	Sample 1a	Extract 1a	LabeledExtract 1a Cy5	Cy5	Hybridization 2
Source 1b	Sample 1b	Extract 1b	LabeledExtract 1b Cy3	Cy3	Hybridization 2
Source 2a	Sample 2a	Extract 2a	LabeledExtract 2a Cy3	Cy3	Hybridization 3
Source 2b	Sample 2b	Extract 2b	LabeledExtract 2b Cy5	Cy5	Hybridization 3
Source 2a	Sample 2a	Extract 2a	LabeledExtract 2a Cy5	Cy5	Hybridization 4
Source 2b	Sample 2b	Extract 2b	LabeledExtract 2b Cy3	Cy3	Hybridization 4

**Table 6:** Representation of the investigation design in Figure 5 as an SDRF.

**Source ID**	**Sample ID**	**Extract ID**	**LabeledExtract ID**	**Label**	**Hybridization ID**
**BS_TKAC_13**	**BSM_TKAC_01m**	**BSM_TKAC_22p**	**BSM_TKAC_22p**	**biotin**	**HFB2002012101A**
BS_TKAC_13	BSM_TKAC_02m	BSM_TKAC_23p	BSM_TKAC_23p	biotin	HFB2002012102A
BS_TKAC_13	BSM_TKAC_03m	BSM_TKAC_24p	BSM_TKAC_24p	biotin	HFB2002012103A
BS_TKAC_13	BSM_TKAC_04m	BSM_TKAC_25p	BSM_TKAC_25p	biotin	HFB2002012104A
BS_TKAC_13	BSM_TKAC_05m	BSM_TKAC_26p	BSM_TKAC_26p	biotin	HFB2002012105A
BS_TKAC_13	BSM_TKAC_06m	BSM_TKAC_27p	BSM_TKAC_27p	biotin	HFB2002012106A
BS_TKAC_13	BSM_TKAC_07m	BSM_TKAC_28p	BSM_TKAC_28p	biotin	HFB2002012107A
BS_TKAC_13	BSM_TKAC_08m	BSM_TKAC_29p	BSM_TKAC_29p	biotin	HFB2002012108A
BS_TKAC_13	BSM_TKAC_09m	BSM_TKAC_30p	BSM_TKAC_30p	biotin	HFB2002012109A
BS_TKAC_13	BSM_TKAC-10m	BSM_TKAC_31p	BSM_TKAC_31p	biotin	HFB2002012110A
**BS_TKAC_14**	**BSM_TKAC_01n**	**BSM_TKAC_22p**	**BSM_TKAC_22p**	**biotin**	**HFB2002012101A**
BS_TKAC_14	BSM_TKAC_02n	BSM_TKAC_23p	BSM_TKAC_23p	biotin	HFB2002012102A
BS_TKAC_14	BSM_TKAC_03n	BSM_TKAC_24p	BSM_TKAC_24p	biotin	HFB2002012103A
BS_TKAC_14	BSM_TKAC_04n	BSM_TKAC_25p	BSM_TKAC_25p	biotin	HFB2002012104A
BS_TKAC_14	BSM_TKAC_05n	BSM_TKAC_26p	BSM_TKAC_26p	biotin	HFB2002012105A
BS_TKAC_14	BSM_TKAC_06n	BSM_TKAC_27p	BSM_TKAC_27p	biotin	HFB2002012106A
BS_TKAC_14	BSM_TKAC_07n	BSM_TKAC_28p	BSM_TKAC_28p	biotin	HFB2002012107A
BS_TKAC_14	BSM_TKAC_08n	BSM_TKAC_29p	BSM_TKAC_29p	biotin	HFB2002012108A
BS_TKAC_14	BSM_TKAC_09n	BSM_TKAC_30p	BSM_TKAC_30p	biotin	HFB2002012109A
BS_TKAC_14	BSM_TKAC-10n	BSM_TKAC_31p	BSM_TKAC_31p	biotin	HFB2002012110A
**BS_TKAC_15**	**BSM_TKAC_01o**	**BSM_TKAC_22p**	**BSM_TKAC_22p**	**biotin**	**HFB2002012101A**
BS_TKAC_15	BSM_TKAC_02o	BSM_TKAC_23p	BSM_TKAC_23p	biotin	HFB2002012102A
BS_TKAC_15	BSM_TKAC_03o	BSM_TKAC_24p	BSM_TKAC_24p	biotin	HFB2002012103A
BS_TKAC_15	BSM_TKAC_04o	BSM_TKAC_25p	BSM_TKAC_25p	biotin	HFB2002012104A
BS_TKAC_15	BSM_TKAC_05o	BSM_TKAC_26p	BSM_TKAC_26p	biotin	HFB2002012105A
BS_TKAC_15	BSM_TKAC_06o	BSM_TKAC_27p	BSM_TKAC_27p	biotin	HFB2002012106A
BS_TKAC_15	BSM_TKAC_07o	BSM_TKAC_28p	BSM_TKAC_28p	biotin	HFB2002012107A
BS_TKAC_15	BSM_TKAC_08o	BSM_TKAC_29p	BSM_TKAC_29p	biotin	HFB2002012108A
BS_TKAC_15	BSM_TKAC_09o	BSM_TKAC_30p	BSM_TKAC_30p	biotin	HFB2002012109A
BS_TKAC_15	BSM_TKAC_10o	BSM_TKAC_31p	BSM_TKAC_31p	biotin	HFB2002012110A
**BS_TKAC_16**	**BSM_TKAC_01q**	**BSM_TKAC_22p**	**BSM_TKAC_22p**	**biotin**	**HFB2002012101A**
BS_TKAC_16	BSM_TKAC_02q	BSM_TKAC_23p	BSM_TKAC_23p	biotin	HFB2002012102A
BS_TKAC_16	BSM_TKAC_03q	BSM_TKAC_24p	BSM_TKAC_24p	biotin	HFB2002012103A
BS_TKAC_16	BSM_TKAC_04q	BSM_TKAC_25p	BSM_TKAC_25p	biotin	HFB2002012104A
BS_TKAC_16	BSM_TKAC_05q	BSM_TKAC_26p	BSM_TKAC_26p	biotin	HFB2002012105A
BS_TKAC_16	BSM_TKAC_06q	BSM_TKAC_27p	BSM_TKAC_27p	biotin	HFB2002012106A
BS_TKAC_16	BSM_TKAC_07q	BSM_TKAC_28p	BSM_TKAC_28p	biotin	HFB2002012107A
BS_TKAC_16	BSM_TKAC_08q	BSM_TKAC_29p	BSM_TKAC_29p	biotin	HFB2002012108A
BS_TKAC_16	BSM_TKAC_09q	BSM_TKAC_30p	BSM_TKAC_30p	biotin	HFB2002012109A
BS_TKAC_16	BSM_TKAC_10q	BSM_TKAC_31p	BSM_TKAC_31p	biotin	HFB2002012110A

The bold highlighting indicates the materials linked to a single hybridization for ease of viewing this example.

**Figure 6:**
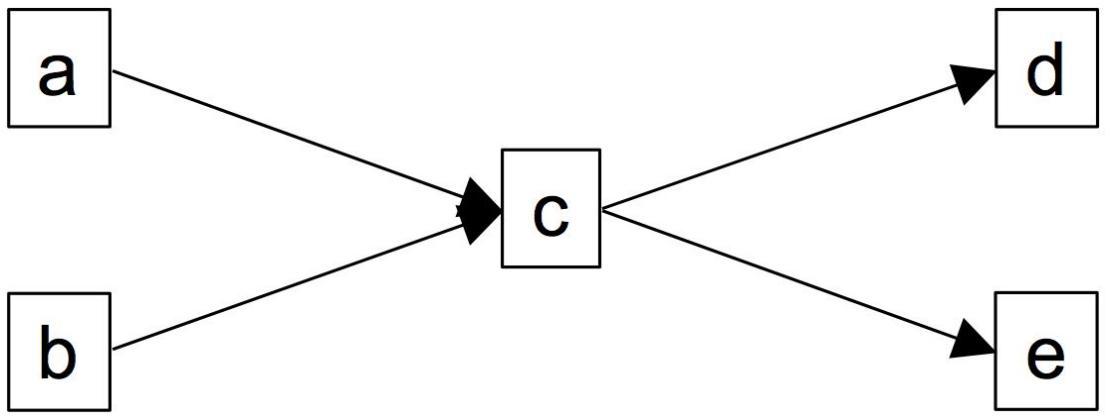
**Graph with four possible paths between nodes**. While four paths are possible between the nodes in this graph [(a → c → d), (a → c → e), (b → c → d), and (b → c → e)], only two full paths, e.g., (a → c → d) and (b → c → e), are required to capture all of the existing relationships between the nodes.

There are several conventions that can be used to make the encoding of DAGs into spreadsheets more concise. First, not every path in a DAG has to be represented on the spreadsheet to encode the DAG unambiguously; it is sufficient to represent every edge only once. For instance, in the graph shown in Figure 6, there are four possible paths (a → c → d), (a → c → e), (b → c → d), and (b → c → e). However, it is enough to present only two full paths, e.g., (a → c → d) and (b → c → e), to represent all the relationships between the nodes in the graph, as shown in the spreadsheet in Table 7. The second 'compaction' rule allows an SDRF spreadsheet to be split vertically on any ID column. More precisely, it is permitted to end an SDRF table at any ID column, and then start a subsequent table with the same column. It is not necessary to duplicate lines for any ID in the second part. For instance, the SDRF in Table 2 can be represented by the two spreadsheets in Tables 8 and 9.

**Table 7:** SDRF representation of the DAG in Figure 6.

**Source layer**	**Mid layer**	**Sink layer**
a	c	d
b	c	e

To describe the IDG in Figure 6, only two of the possible four paths need be shown; redundant edges in the graph may be omitted.

**Table 8:** Representing SDRF from Table 2 by a set of two SDRF files: first spreadsheet.

**Sample ID**	**Characteristics [Organism]**	**Characteristics [OrganismPart]**	**Protocol REF**	**Extract ID**	**Protocol REF**	**Label**	**Hybridization ID**
liver 1	Homo sapiens	liver	P-XMPL-1	Extract 1	P-XMPL-3	Cy3	Hyb 1
liver 2	Homo sapiens	liver	P-XMPL-1	Extract 2	P-XMPL-3	Cy3	Hyb 1
kidney 1	Homo sapiens	kidney	P-XMPL-1	Extract 3	P-XMPL-3	Cy3	Hyb 2
kidney 2	Homo sapiens	kidney	P-XMPL-1	Extract 4	P-XMPL-3	Cy3	Hyb 2
brain 1	Homo sapiens	brain	P-XMPL-1	Extract 5	P-XMPL-3	Cy3	Hyb 3
brain 2	Homo sapiens	brain	P-XMPL-1	Extract 6	P-XMPL-3	Cy3	Hyb 3
				Extract reference	P-XMPL-3	Cy5	Hyb 1
				Extract reference	P-XMPL-3	Cy5	Hyb 2
				Extract reference	P-XMPL-3	Cy5	Hyb 3

Such splitting of an SDRF spreadsheet can be done on any ID column, which becomes the last column in the first spreadsheet (this table) and is repeated as the first column in the second spreadsheet (Table 9).

**Table 9:** Representing SDRF from Table 2 by a set of two SDRF files: second spreadsheet.

**Hybridization ID**	**ArrayDesign REF**	**ArrayData URI**	**DerivedArrayData Matrix URI**
Hyb 1	SMD-10K	1.txt	FGDM.txt
Hyb 2	SMD-10K	2.txt	FGDM.txt
Hyb 3	SMD-10K	3.txt	FGDM.txt

See the legend to Table 8 for discussion. Because each Hybridization ID only needs to be represented once, the second partial spreadsheet has three rows instead of nine (discounting the header row).

For a detailed description of rules for encoding an arbitrary investigation design graph as an SDRF file, see the MAGE-TAB online documentation [22].

#### Data files

The MAGE-TAB specification requires that raw data files are provided as binary or ASCII files in their native formats, such as Affymetrix CEL files, Agilent TXT files, or GenePix GPR files, whereas processed data files may be communicated in tab-delimited text format as *data matrix *files. Normally, a MAGE-TAB document will have one data matrix where rows typically represent genes (though they may also represent other biological entities, such as exons or genomic locations), and columns typically represent samples or experimental conditions. One can think of such a matrix as containing the data that are typically published as supplementary information for a given paper and on which the author would perform analyses such as clustering.

The main feature of data matrices, that distinguishes them from arbitrary data files, is that columns in such matrices have references to ID objects in SDRF files, for instance to particular raw data files or particular samples. This enables mapping from biomaterials and their characteristics (especially experimental factor values) to individual processed data columns by following the edges in the investigation design graph. Syntactically, each data matrix file has two header rows, as shown in Table 10. The first header row contains references to ID objects in an SDRF file. All the IDs should come from one particular column in the SDRF. That is, each column in the data matrix is marked by unique IDs from a particular column in the SDRF. The second row contains the names of the quantitation types, such as 'signal', 'p-value', or 'log_ratio(Cy3/Cy5)' (from the MAGE-TAB perspective, these are simply labels that do not have to have a particular meaning, but normally should be defined in the data processing protocol). An example is shown in Table 10.

**Table 10:** An example of a data matrix.

**ArrayData REF**	Data1.cel	Data1.cel	Data2.cel	Data2.cel	Data3.cel	Data3.cel
**Reporter REF**	signal	p-value	signal	p-value	signal	p-value

Gene 1	x11	p11	x21	p21	x31	p31
Gene 2	x12	p12	x22	p22	x32	p32
Gene 3	x13	p13	x23	p23	x33	p33
...	...	...	...	...	...	...
Gene n	x1n	p1n	x2n	p2n	x3n	p3n

The first row gives references to objects in an SDRF file, for instance to ArrayData URIs in the SDRF in Table 1. The second row specifies the names of the quantitation types that are represented in each column. The first column gives the names of the biological objects these 'expression' measurements relate to, for instance the IDs of the reporters or composite elements in the ADF file or files describing the design of array(s) on which these measurements have been performed. Alternatively, this column may contain identifiers from public sequence databases, or chromosome coordinates from a specified genome build.

Using this mapping each column in the summary data matrix can be automatically and concisely annotated by the most important characteristics, such as experimental factor values (see next section).

## Results and Discussion

### Experimental factors and their values

*Experimental factors *and experimental factor values are important concepts in MIAME. The experimental factors are the principal variables in the investigation, for instance "time" in time series investigations, "dose" in dose response investigations, "compound" in compound treatment investigations, or "disease state" (normal or otherwise) in disease studies. The same investigation may have several experimental factors; for example, compound, dose and time may all be experimental factors in a dose response investigation in which several compounds are added to the samples over a time course. Experimental factors and their values can appear in the SDRF file in any column (Table 11), and are annotated as such by being listed in the IDF file. For example, the IDF linked to the SDRF in Table 11 would include the MGED Ontology term "Time" in its list of experimental factors (see Table 3), indicating that the "ParameterValue [Time]" column represents an experimental variable. Biological replicates are represented by distinct biological sources, grouped together by common experimental factor values. In contrast, technical replicates are represented by branching of the investigation design graph at intermediate steps of the experimental processing.

The experimental factor values are the values of the respective experimental factors in a particular sample. For instance, in a time series the values are the time points at which each measurement was taken.

Experimental factor values provide a means of annotating investigations concisely – the most important experimental variables are clearly and accessibly defined. Moreover, one can easily represent biological replicates: these are samples which have different sources, but exactly the same values for all experimental factors. By propagating the factor values down to data columns in the processed data, one can annotate data concisely. For instance, if we have two experimental factors compound and dose, each of which have two possible values, e.g. compounds c1 and c2, and low dose and high dose, then the data columns will be annotated by combinations of these values: (c1, low), (c2, low), (c1, high), (c2, high).

**Table 11:** Experimental factor values example. The Characteristics categories used in column headings (i.e., the terms in square brackets) are taken from the MGED Ontology "BioMaterialCharacteristics" class [26]. The values contained in the body of these columns may be either free text, or terms from an ontology as indicated by an "OI" tag in the column heading (relating to the MAGEv2 concept "OntologyIndividual"). For example, the "OI:nci_meta" tag indicates that terms are taken from the NCI Metathesaurus [27]. The sources for these database tags ("nci_meta", "CTO") are defined in the IDF, as shown in Table 3. Biological replicates are indicated by shared experimental factor values ("Time" in this example; the columns containing experimental factors would be specified in the accompanying IDF). Most of the protocols have been omitted for brevity. Please see the detailed MAGE-TAB specification document [22] for more information.

**Source ID**	**Characteristics [CellLine]**	**Characteristics [CellType] OI:CTO**	**Characteristics [DiseaseState] OI:nci_meta**	**Characteristics [Organism] OI:ncbitax**	**Protocol REF**	**ParameterValue [Time]**	**Unit [TimeUnit] OI:MO**	**Hybridization ID**	**ArrayDesign REF**
ARP1-0h	MOLT4	T cell	acute lymphoblastic leukemia	Homo sapiens	P-XMPL-3	0	hours	H_ARP1-0h	A-AFFY-33
ARP2-0h	MOLT4	T cell	acute lymphoblastic leukemia	Homo sapiens	P-XMPL-3	0	hours	H_ARP2-0h	A-AFFY-33
ARP3-0h	MOLT4	T cell	acute lymphoblastic leukemia	Homo sapiens	P-XMPL-3	0	hours	H_ARP3-0h	A-AFFY-33
ARP1-2h	MOLT4	T cell	acute lymphoblastic leukemia	Homo sapiens	P-XMPL-3	2	hours	H_ARP1-2h	A-AFFY-33
ARP2-2h	MOLT4	T cell	acute lymphoblastic leukemia	Homo sapiens	P-XMPL-3	2	hours	H_ARP2-2h	A-AFFY-33
ARP3-2h	MOLT4	T cell	acute lymphoblastic leukemia	Homo sapiens	P-XMPL-3	2	hours	H_ARP3-2h	A-AFFY-33
ARP1-4h	MOLT4	T cell	acute lymphoblastic leukemia	Homo sapiens	P-XMPL-3	4	hours	H_ARP1-4h	A-AFFY-33
ARP2-4h	MOLT4	T cell	acute lymphoblastic leukemia	Homo sapiens	P-XMPL-3	4	hours	H_ARP2-4h	A-AFFY-33
ARP3-4h	MOLT4	T cell	acute lymphoblastic leukemia	Homo sapiens	P-XMPL-3	4	hours	H_ARP3-4h	A-AFFY-33
ARP1-6h	MOLT4	T cell	acute lymphoblastic leukemia	Homo sapiens	P-XMPL-3	6	hours	H_ARP1-6h	A-AFFY-33
ARP2-6h	MOLT4	T cell	acute lymphoblastic leukemia	Homo sapiens	P-XMPL-3	6	hours	H_ARP2-6h	A-AFFY-33
ARP3-6h	MOLT4	T cell	acute lymphoblastic leukemia	Homo sapiens	P-XMPL-3	6	hours	H_ARP3-6h	A-AFFY-33
ARP1-8h	MOLT4	T cell	acute lymphoblastic leukemia	Homo sapiens	P-XMPL-3	8	hours	H_ARP1-8h	A-AFFY-33
ARP2-8h	MOLT4	T cell	acute lymphoblastic leukemia	Homo sapiens	P-XMPL-3	8	hours	H_ARP2-8h	A-AFFY-33
ARP3-8h	MOLT4	T cell	acute lymphoblastic leukemia	Homo sapiens	P-XMPL-3	8	hours	H_ARP3-8h	A-AFFY-33
ARP1-10h	MOLT4	T cell	acute lymphoblastic leukemia	Homo sapiens	P-XMPL-3	10	hours	H_ARP1-10h	A-AFFY-33
ARP2-10h	MOLT4	T cell	acute lymphoblastic leukemia	Homo sapiens	P-XMPL-3	10	hours	H_ARP2-10h	A-AFFY-33
ARP3-10h	MOLT4	T cell	acute lymphoblastic leukemia	Homo sapiens	P-XMPL-3	10	hours	H_ARP3-10h	A-AFFY-33
ARP1-12h	MOLT4	T cell	acute lymphoblastic leukemia	Homo sapiens	P-XMPL-3	12	hours	H_ARP1-12h	A-AFFY-33
ARP2-12h	MOLT4	T cell	acute lymphoblastic leukemia	Homo sapiens	P-XMPL-3	12	hours	H_ARP2-12h	A-AFFY-33
ARP3-12h	MOLT4	T cell	acute lymphoblastic leukemia	Homo sapiens	P-XMPL-3	12	hours	H_ARP3-12h	A-AFFY-33

### Applications of MAGE-TAB

MAGE-TAB can be used in either of two ways:

1. Creating MAGE-TAB documents using spreadsheet templates provided in the main specification document [22] or creating them 'from scratch';

2. Developing MAGE-TAB export or import functionality for microarray databases or tools.

Details of the second use depend on the particular structure of the database or tool; as this is mostly aimed at software developers and professional bioinformaticians it will not be discussed here. The first approach may be used by biologists to keep track of data, to submit the data to either public or private repositories, or to exchange data. Use of existing template SDRF documents can simplify this approach. The MAGE-TAB specification document provides templates for several 'standard' experimental designs for one- and two-channel investigations including:

1. simple replicated design;

2. replicated design with technical replicates;

3. replicated design with pooling;

4. replicated designs for dual channel investigations;

5. dual channel replicated designs with dye swap;

6. dual channel replicated designs with a reference sample;

7. dual channel replicated design with a reference and dye swap;

8. dual channel replicated design with a pooled reference;

9. loop design;

10. loop design with dye swap;

11. time series investigations.

This list is by no means exhaustive; in fact, MAGE-TAB does not prescribe any particular investigation design. Rather, the purpose of this template collection is to help users to create an SDRF file for their particular design. IDF and ADF files are quite straightforward to create; moreover, for experiments that are done on standard arrays, such as Affymetrix or Agilent, biologists do not need to create the ADF files, as they are provided by the manufacturers and public databases.

## Conclusion

MAGE-TAB is designed to serve as a format for data collection, presentation, and communication. Some may argue that the data communication format should be separated from the collection and presentation format, on the grounds that the data communication format is designed solely for computers, whereas data collection and presentation depend upon human-computer interactions. This convention has been used in MAGE-ML with some success. However, the separation of these formats can only work well if there are good tools to interconvert the data collection and communication formats. Because tool development has proven to be expensive and time consuming, a format that is simple enough to be usable without any specialized tools is needed at present. MAGE-TAB serves as such a format.

A valid caveat regarding spreadsheet-based formats relates to the limitations of currently-available spreadsheet software. At present, the maximum number of rows allowable in a Microsoft Excel or OpenOffice spreadsheet is 65,536; it is anticipated that many array designs will exceed this number of features, rendering such software packages less useful for constructing ADF files. In addition, these software packages often implement automatic date format and floating point conversions which can silently alter values entered into a spreadsheet to render them invalid [24]. Care must therefore be taken when using these programs, for instance by formatting the entire spreadsheet as plain-text prior to entering any data. 

Some would argue that XML-based formats are more appropriate for data exchange than tabular formats. However, this is not universally true – XML is most appropriate for representing tree-like structures, whereas investigation design graphs are DAGs. It can be quite cumbersome to represent a DAG in an XML-based format. Our observation that the DAGs corresponding to investigation graphs are regular, layered and have a small node degree (except for the reference nodes), has enabled us to find a natural way to represent such DAGs in a spreadsheet format. As far as array designs are concerned, their representation as spreadsheets is straightforward since such data are naturally encoded in a tabular format. 

Moreover, since the level of granularity in MAGE-TAB is consistent with that of MIAME, it also offers a formal model for representing MIAME-compliant data that is simpler than the full MAGE object model. We propose that MAGE-TAB becomes a platform-specific implementation of the 'simple layer' of MAGE. As such, MAGE-TAB will be more limited in expressivity than MAGE-ML. For example, protocols are described as free text with optional parameters, rather than as a series of discrete steps. 

The ArrayExpress database is planning to accept submissions in the finalized MAGE-TAB format as soon as practically possible. In effect, a prototype called 'Tab2MAGE' [25] which uses spreadsheets similar to the MAGE-TAB format has already been implemented and proven to work. (ArrayExpress will also continue to accept submissions in MAGE-ML, and will accept MAGE-ML v2 when it is available). ArrayExpress will also provide data export in MAGE-TAB format. In the next 12–24 months we will gather feedback on how well this format works in practice, and make the necessary revisions, as part of the MAGEv2 development process. Nevertheless, we expect that the format will be stable, and even if some changes are needed in the future, we aim to provide software for interconverting the formats. 

In conclusion, we note that MAGE-TAB has been designed to address the needs of the microarray community, by providing a simple format for representing and communicating MIAME-compliant data in a structured way with minimal investment.

## Authors' contributions

All authors contributed to the design of MAGE-TAB through participating in one or more of the five MAGE workshops held during 2005 and 2006, or via e-mail discussions. TR designed and implemented the Tab2MAGE prototype, and wrote the final draft of the manuscript. PR-S designed the ADF component of MAGE-TAB. AF, EH and HP participated in testing and evaluation of the Tab2MAGE prototype, and documented the core use cases for MAGE-TAB. CB and US helped to design the IDF component. PS was the main organizer of the MAGE workshops, and was leading the critical discussions. AF implemented a visualization tool for IDGs and analyzed their structure in ArrayExpress. PS, HC, CS, JW, RI and DM contributed also to different drafts of the manuscript. JL, KP, MM, PW and US helped in relating the format to MAGE-ML and the MGED Ontology. AB initiated and coordinated the design process for MAGE-TAB, and wrote the first draft of the manuscript. All authors read and approved the final manuscript.
